# The Value of Journalism to Hospitals

**Published:** 1920-05-08

**Authors:** C. O. Hawthorne


### THE VALUE OF JOURNALISM TO HOSPITALS.

**By C. O. Hawthorne, M.D.**

The announcement of the death of Sir Henry Burdett will awake sorrow and regret in numerous and varied quarters. His strong personality and abounding energy touched life at many points, and his keen sympathy with effort and achievement, and his ability to inspire and direct, made him a recognised leader in each of the several fields where his activities displayed themselves. It is thus inevitable that workers of various orders must to-day be conscious that there has gone from their midst both a far-seeing and experienced guide and a loyal and helpful comrade. Both to causes and to men he gave himself without limitation and without stint, and the index of their loss is the measure of his generous and wholehearted service. His name is written deeply in large enterprises and also in individual hearts, and what he was and what he did will long be cherished as an abiding and grateful memory.

To readers of these pages Sir Henry Burdett will be known principally from his long and unfailing interest in hospital work. Here, indeed, was the passion of his life. In his very early days he made himself a student of the hospital system, and for many years he has occupied a recognised and commanding position in the hospital world. No department of the work was outside his knowledge or outside his interest, and not a few of them are the better for his frank and authoritative criticism. For all sincere effort he had a deep and sympathetic regard, but inefficiency in matters which he judged of high importance he could not tolerate, and he found appropriate words for his censure. One of his early convictions was the need for the improvement of the status of members of the administrative staffs of the hospitals and for attracting the interests of the public to hospital work and service generally. It was this conviction which led him to found a journal having for its aims the improvement of all branches of hospital work and the better organisation of hospital workers. By extending knowledge of the work and workers he hoped, also, to attract the attention of the public and to urge the duty of all to do something in the cause of the sick and suffering. Never was he tired of presenting the hospital as an opportunity for the cultivation of sympathy and helpfulness, and he claimed from the community not merely money, but personal service, on its behalf. This text he preached again and again in leading articles, in letters to the Press, and in speeches from a hundred platforms both at home and abroad. Whoever had some practical proposal relative to hospital work found in him a ready listener, and appeals for counsel and help from various parts of the country were with him almost a daily experience. No hospital, however obscure or remote, was outside the range of his interest, and his editorial columns were open to every cry for justice and aid. It was this attitude of mind which largely determined his firm adhesion to the principle of the voluntary hospitals. To substitute for the voluntary system institutions under bureaucratic control seemed to him, apart from any question of efficiency, the loss of an educative and humanising influence in the life of the country. Both patients and public would, he held, be the poorer by the removal of an opportunity for the recognition of claims which appeal to the best instincts of human emotion. Difficulties, financial and otherwise, he either would not admit or would resolve to overcome. The results of his faith and courage are seen in the successful enterprises recently undertaken in many provincial centres, and but for his illness he would have carried a crusade on behalf of the hospitals through the length and breadth of the land.

There can be no doubt that by his journalistic and other activities Sir Henry Burdett accomplished a large part of the purpose which he set before himself at the outset. His weekly pages formed a meeting-place for all hospital activities, and in this way was cultivated a rising standard of efficiency and a community of interest and goodwill. Hospital work was introduced to the public as one in which thoroughness, business capacity, enterprise, and originality may find a legitimate opportunity, and where there was no room for dawdlers or incompetents. The nursing and administrative staffs in particular owe much to this advocacy, and unquestionably public opinion has responded, at least in some measure, to the appeal. Sir Henry certainly provided criticism when he thought it necessary, but for the most part his argument was the dignity of hospital work and the claim that to the sick and unfortunate nothing less than the best should be given.

There is another aspect of the hospital system on which Sir Henry Burdett set great store—namely, the training of medical students and the cultivation of research. As he repeatedly urged, these activities make every member of the community a debtor to the hospitals. The standards of professional efficiency and professional honour are transmitted through the medical schools to succeeding generations of the profession, with advantage to the public beyond the definition of words; while the application of some of the best minds to medical problems old and new secures those additions to knowledge which make successful progress in medical and surgical treatment possible. To take but a single example, let it be remembered that it was in the wards of a voluntary hospital in Glasgow that Lister, as a visiting surgeon, devised and developed the antiseptic system of surgery.

That a system marked by so much achievement should be put in peril or should be allowed to founder for want of funds was in Sir Henry's judgment unthinkable. The public must be educated and informed, and this done, there could be no doubt of the issue. Such was his unwearied aim,

**The Value of Journalism to Hospitals.—(*continued*).**

and his faith was equal to the task. Unfortunately his strength failed before full victory had been attained, but it is certain that in so far as public opinion has been led to recognise the value and virtues of the voluntary-hospital system the result is very largely due to the voice and pen of Sir Henry Burdett. He believed in this system wholeheartedly, and he gave himself freely to its support. Far from neglecting the emotional and humanising appeal of the voluntary hospitals, he placed this influence in the foreground. People should serve and help the hospitals, he urged, not only for the welfare of the patients, but also for their own welfare. Yet not less emphatic was he for the cultivation of efficiency and economy and business methods. For a well-meaning charity which was shaky in its accounts he had little patience. And to the public he would say quite plainly that it was not only their duty, but also their interest, to support a system which gave them a well-trained medical profession and secured for all classes highly expert services when the need for these is imperative. All these convictions were the outcome of a long experience and careful study. Sir Henry held them consistently, and they ruled, not only his mind but also his actions. Whatever he could do to secure their recognition and adoption he did, and even those who differed from him in opinion will remember his abounding zeal and generous service and will gladly agree that his life was one of sincere devotion to great ends.

